# Overcoming the senescence‐associated secretory phenotype (SASP): a complex mechanism of resistance in the treatment of cancer

**DOI:** 10.1002/1878-0261.13042

**Published:** 2021-07-12

**Authors:** Cecilia R. Chambers, Shona Ritchie, Brooke A. Pereira, Paul Timpson

**Affiliations:** ^1^ Garvan Institute of Medical Research The Kinghorn Cancer Centre Sydney NSW Australia; ^2^ Faculty of Medicine St. Vincent's Clinical School University of New South Wales Sydney NSW Australia

**Keywords:** cancer, cellular senescence, senescence‐associated secretory phenotype, therapy‐induced senescence

## Abstract

Senescence is a cellular state in which cells undergo persistent cell cycle arrest in response to nonlethal stress. In the treatment of cancer, senescence induction is a potent method of suppressing tumour cell proliferation. In spite of this, senescent cancer cells and adjacent nontransformed cells of the tumour microenvironment can remain metabolically active, resulting in paradoxical secretion of pro‐inflammatory factors, collectively termed the senescence‐associated secretory phenotype (SASP). The SASP plays a critical role in tumorigenesis, affecting numerous processes including invasion, metastasis, epithelial‐to‐mesenchymal transition (EMT) induction, therapy resistance and immunosuppression. With increasing evidence, it is becoming clear that cell type, tissue of origin and the primary cellular stressor are key determinants in how the SASP will influence tumour development and progression, including whether it will be pro‐ or antitumorigenic. In this review, we will focus on recent evidence regarding therapy‐induced senescence (TIS) from anticancer agents, including chemotherapy, radiation, immunotherapy, and targeted therapies, and how each therapy can trigger the SASP, which in turn influences treatment efficacy. We will also discuss novel pharmacological manipulation of senescent cancer cells and the SASP, which offers an exciting and contemporary approach to cancer therapeutics. With future research, these adjuvant options may help to mitigate many of the negative side effects and protumorigenic roles that are currently associated with TIS in cancer.

## Graphical abstract

In response to therapy or other stimuli, cancer cells can undergo cellular senescence, a stress response leading to cell cycle arrest. An unintended consequence of cellular senescence is a perturbed, pro‐inflammatory cancer cell secretome, the senescence‐associated secretory phenotype (SASP). Here, we explore the role of SASP in promoting tumorigenesis and treatment resistance, with particular focus on mechanisms of therapy‐induced senescence (TIS).

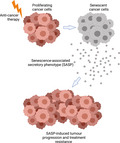

**Abbreviations**
ADAM17: ADAM (a disintegrin and metalloproteinase) metallopeptidase domain 17
ALCAM: activated leukocyte cell adhesion molecule
ALK: anaplastic lymphoma kinase
AMPK: 5' AMP‐activated protein kinase
ATM: ataxia telangiectasia mutated
BCL‐2: B‐cell lymphoma 2
BETi: bromodomain and extraterminal protein inhibitors
BH3: Bcl‐2 homology 3
BMI1: B lymphoma Mo‐MLV insertion region 1 homolog
BRAF: v‐raf murine sarcoma viral oncogene homolog B1
CAF‐T: chimeric antigen receptor T cells
CDC7: cell division cycle kinase 7
CDKs: cyclin‐dependent kinases
C‐EBP: CCAAT/enhancer‐binding protein
CHK2: checkpoint kinase 2
CSCs: cancer stem cells
DCA: deoxycholic acid
DCs: dendritic cells
DDR: DNA damage response
DIPG: diffuse intrinsic pontine glioma
DNA: deoxyribonucleic acid
DOX: doxorubicin
EGF/R: epidermal growth factor/receptor
EMT: epithelial‐to‐mesenchymal transition
EOC: epithelial ovarian cancer
EV: extracellular vesicles
FBP1: fructose‐1,6‐bisphosphatase
FGF/R: fibroblast growth factor/receptor
GCV: ganciclovir
GM‐CSF: granulocyte‐macrophage colony‐stimulating factor
H3K27M: substitution mutation of lysine for methionine at position 27 in histone H3
HCC: hepatocellular carcinoma
HGF: hepatocyte growth factor
HGSC: high‐grade serous carcinoma
HNSCC: head and neck squamous cell carcinoma
HSCs: hepatic stellate cells
ILs: interleukins
IRE1: inositol‐requiring enzyme 1
JAK‐STAT: Janus kinase/signal transducer and activator of transcription
M‐CSF: macrophage colony‐stimulating factor
MMP: matrix metalloproteinase
mTOR: mammalian target of rapamycin
NAMPT: nicotinamide phosphoribosyltransferase
NASP: NFκB‐driven SASP
NFκB: nuclear factor kappa‐light‐chain‐enhancer of activated B cells
NK: natural killer (cells)
p38 MAPK: p38 mitogen‐activated protein kinase
PARP: poly(ADP‐ribose) polymerase
PASP: p53‐driven SASP
PD‐1: programmed cell death protein 1
PDGFa/b: platelet‐derived growth factor subunit A/B
PTBP1: polypyrimidine tract‐binding protein 1
Pten: phosphatase and tensin homolog
PyMT: polyomavirus middle T antigen
QoL: quality of life
Rb: retinoblastoma
RNA: ribonucleic acid
ROCK: Rho‐associated coiled‐coil containing kinases
ROS: reactive oxygen species
SASP: senescence‐associated secretory phenotype
scRNASeq: single‐cell RNA sequencing
siRNA: short interfering RNA
TGFB: transforming growth factor beta
TIMP1: tissue inhibitor of metalloproteinase 1
TIS: therapy‐induced senescence
TME: tumour microenvironment
TNBC: triple‐negative breast cancer
TOP1: topoisomerase I
TOP1cc: TOP1‐DNA cleavage complexes
uPAR: urokinase‐type plasminogen activator receptor
VEGF/R: vascular growth factor/receptor

## 1 Introduction

**Fig. 1:**
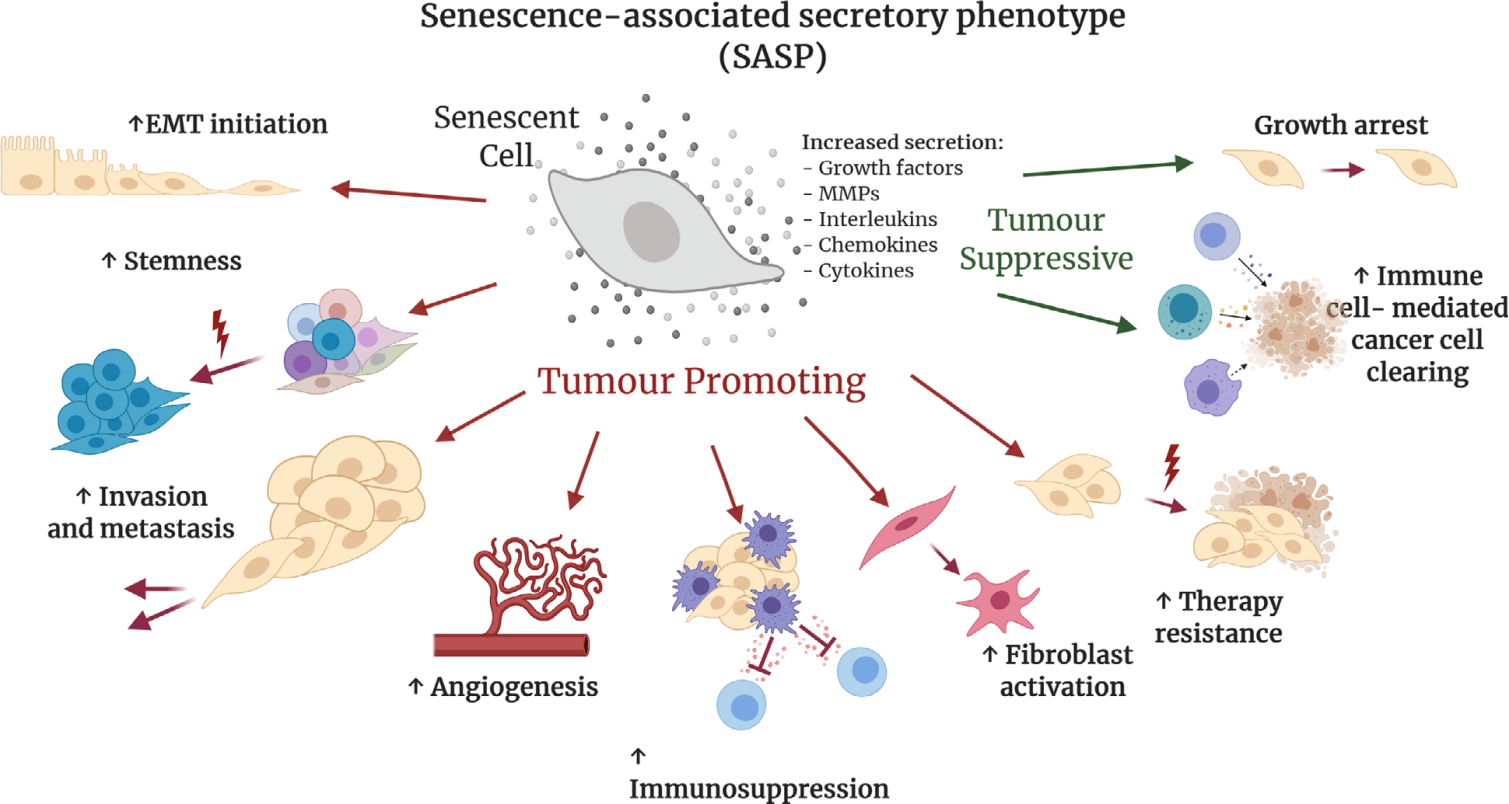
Senescence‐associated secretory phenotype (SASP) exhibits both tumour‐promoting and tumour‐suppressing roles. Senescent cells secrete a number of factors. including growth factors, MMPs, interleukins, chemokines and cytokines, which can play many complex roles in the tumour microenvironment (TME). The red arrows highlight tumour‐promoting roles, including increases in epithelial‐to‐mesenchymal (EMT) initiation, cancer cell stemness, invasion and metastasis, angiogenesis, immunosuppression, fibroblast activation and therapy resistance. The green arrows highlight tumour‐suppressive roles of SASP, including cell cycle arrest and an increase in immune surveillance resulting in improved cancer cell clearing by the immune system.

Cellular senescence is an important mechanism that has evolved to protect tissues from dangerous overproliferation of cells and therefore plays a tumour‐suppressive role [1]. In malignancy, cancer cells begin to proliferate uncontrollably, and somatic mutations are acquired at a greater frequency than in normal tissue [2]. Therefore, in many circumstances, cellular senescence is a protective process that safeguards the tissue from the development of cancer by permanently halting the cell cycle, inhibiting proliferation and preventing the propagation of deleterious genetic mutations. For this reason, anticancer therapies often aim to impede cancer progression by inducing cell cycle arrest [3]. Despite this concept being initially advantageous in reducing tumour burden, a key setback in overall patient outcome is the eventual therapy resistance and/or relapse that many patients endure. Although targeted cancer cells have stopped proliferating, senescent cells remain metabolically active and can have the unintended side effect of developing an altered cellular secretome, which is associated with local inflammation, extracellular changes and increased growth factor activity [4]. These secretions, collectively known as the senescence‐associated secretory phenotype (SASP), are a dynamic, cell type‐dependent phenomenon where senescent cells secrete high levels of cytokines, chemokines, proteases, growth factors and extracellular vesicles (EVs), all of which are typically pro‐inflammatory. This response is due to a persistent cellular stressor that can elicit a DNA damage response (DDR), which can be induced in both cancer cells and nontransformed cells of the tumour microenvironment (TME) [5] and can be both pro‐ or antitumorigenic. For example, the SASP can promote epithelial–mesenchymal transition (EMT) initiation [6, 7], stemness induction [8, 9], local tissue invasion [10], angiogenesis [11], activation of fibroblasts [12], immunosuppression [13, 14], enhanced metastasis [15] and therapy resistance [16, 17, 18, 19] (Fig. 1, see red arrows). Conversely, the SASP has also been shown to aid chemotherapy delivery [20] as well as enhance clearing of senescent cancer cells via recruitment of immune cells such as natural killer (NK) cells, macrophages and cytotoxic T cells [13, 21, 22, 23, 24], therefore impeding tumour progression (Fig. 1, see green arrows).

Due to cancer therapeutics generally being administered systemically, a problem arises when surrounding nontransformed cells become senesced as a response to the cytotoxic agent. As a result, an accumulation of senescent cells can cause the onset of secondary disease such as fibrosis‐driven disorders, cardiothoracic diseases and neurodegenerative disease [25]. The SASP can be further reinforced via autocrine signalling [26, 27], while also spreading to adjacent cells and tissues in a long‐range paracrine manner [28], including via EVs [29]. Recently, there have been attempts to eliminate SASP‐expressing cells to enhance anticancer therapies by using senescence‐targeting drugs (known as ‘senolytics’ or ‘senostatics’), which hold some promise for overcoming this diverse and complex resistance mechanism. Here, we will describe the latest discoveries in the context of therapy‐induced senescence (TIS) and the SASP, and its dynamic role in both promoting and mitigating tumour development and progression.

## 2 Diversity of SASP induction

Senescence can occur from several sources of cellular stressors, including genomic or telomeric injury, epigenomic disturbances, oxidative stress and changes to oncogene or tumour suppressor expression [30]. These processes can activate the tumour suppressor pathways p53 and retinoblastoma (Rb), which can then cause increased p16^INK4A^ expression, triggering cell cycle arrest [31]. Several downstream pathways involved in DNA damage, cell cycle arrest and inflammation can then become activated, inducing the SASP in these cells. These pathways include ATM [32], CHK2 [33], p38 MAPK [34, 35], CCAAT/enhancer‐binding proteins (C/EBP) [27], NFκB [21], mTOR [36] and JAK‐STAT signalling [37], which have been summarised extensively by Faget, Ren [31]. However, senescence can also be triggered by direct overexpression of p16^INK4A^, which does not produce an inflammatory SASP (normally mediated by NFκB), for reasons that are still being elucidated [38, 39, 40]. Due to the diverse nature of secreted factors and involvement of several prominent transcriptional pathways, biomarkers for SASP‐expressing cells have been difficult to identify [41]. Despite this, a recent study was able to identify 55 genes aligned with a common ‘senescence‐associated’ signature, albeit with high levels of transcriptional heterogeneity between senescent cell types [41]. In general, the SASP is considered highly cell type‐dependent and diverse in secreted factors; however, there are several SASP markers that are shared by most SASP‐expressing cells. These markers include (but are not limited to) growth factors (vascular endothelial growth factor (VEGF), platelet‐derived growth factor (PDGF), hepatocyte growth factor (HGF)), interleukins (IL‐1α, IL‐6, IL‐8, IL‐10, IL‐13, IL‐15), matrix metalloproteinases (MMP3, MMP9) and cytokines/chemokines (CXCL1, CXCL2, CXCL5, CXCL11, CXCL12, CCL2, CCL20); however, it is important to note that none of these markers are truly specific to the phenotype alone [31]. That is, these cellular secretions often overlap with common markers of cell type or cellular activation, as well as inflammation and proliferation [31]. Efforts are now being made to further profile the SASP of different cells by characterising the specific factors being secreted in order to develop better biomarkers. For example, Basisty *et al*. (2020) have recently developed the ‘SASP Atlas’, which is the first proteomic‐based database for soluble SASP‐related factors and SASP factors contained in exosomal cargo [42]. This resource offers insight into the diversity of the SASP secreted factors, which is dependent on both stressor that induces the SASP and also cell type [42]. Therefore, it is important to consider that the specific features of the SASP are determined not only by the senescent cell type and tissue, but also by what caused senescence to occur.

Interestingly, recent work has highlighted that alterations in cellular metabolism may play a central role in SASP activation, thereby promoting cancer development and progression. For example, Yoshimoto and colleagues (2013) showed that obesity can induce the SASP in hepatic stellate cells (HSCs) via increased levels of deoxycholic acid (DCA), a metabolite that can cause DNA damage [43]. These SASP‐expressing HSCs then produced increased levels of inflammatory cytokines, enhancing the development of carcinogen‐stimulated hepatocellular carcinoma (HCC) in mice [43]. More recently, Li *et al*. (2020) reported that hepatocyte‐specific deletion of fructose‐bisphosphatase 1 (FBP1), a rate‐limiting enzyme in the process of gluconeogenesis, results in a fatty liver as well as activation of HSCs, which exhibit the SASP [44]. When treated with drugs that can kill senescent cells, such as dasatinib or ABT‐263, this effect was reversed, highlighting FBP1 as a potential novel liver cancer tumour suppressor [44]. Similarly, a preclinical study by Dorr *et al*. (2013) reported that senescent lymphomas were shown to utilise glucose at a much higher rate due to the presence of the SASP [45]. In this study, the senescent cells relied heavily on glucose utilisation as a response to cope with the macromolecular stress induced by the SASP [45]. When glucose utilisation was inhibited, tumours regressed more successfully following chemotherapy treatment [45]. Moreover, it has been shown that a high level of nicotinamide phosphoribosyltransferase (NAMPT), a rate‐limiting enzyme in the NAD^+^ pathway, can determine the strength of the pro‐inflammatory SASP [46]. This study by Nacarelli *et al*. (2019) showed that a high NAD^+^/NADH ratio could regulate AMPK signalling and downstream NFκB signalling [46]. This, in turn, could increase the strength of the pro‐inflammatory SASP, thereby enhancing mitochondrial respiration and glycolytic processes [46]. Considering that NAMPT inhibitors are currently in clinical trials (reviewed in [47, 48]), this study offers further insight into how the molecular mechanisms of the SASP can be clinically targeted in the context of dysregulated cellular metabolism. Interestingly, there is also evidence that cancer cells have the ability to utilise unusual nutrient sources when in a senescent state. For example, Tonnessen‐Murray *et al*. (2019) reported that breast cancer cells rendered senescent with doxorubicin (Dox) treatment can completely engulf neighbouring cells via a phagocytosis‐like phenomenon to aid their survival [49]. This study highlights the resourcefulness of senesced cells, where they can exploit surrounding cells as a source of nutrients to survive during dormancy, allowing them to potentially relapse at a later stage.

Overall, mainstream cancer treatment modalities such as chemotherapy, radiation, immunotherapy, and targeted therapies aim to kill cancer cells; however, sometimes only senescence is achieved. Activating cellular senescence has been shown to significantly modulate the cancer secretome via activation of a therapy‐induced SASP, which is discussed in detail below.

### 2.1 Chemotherapy and radiation induce the SASP

**Fig. 2:**
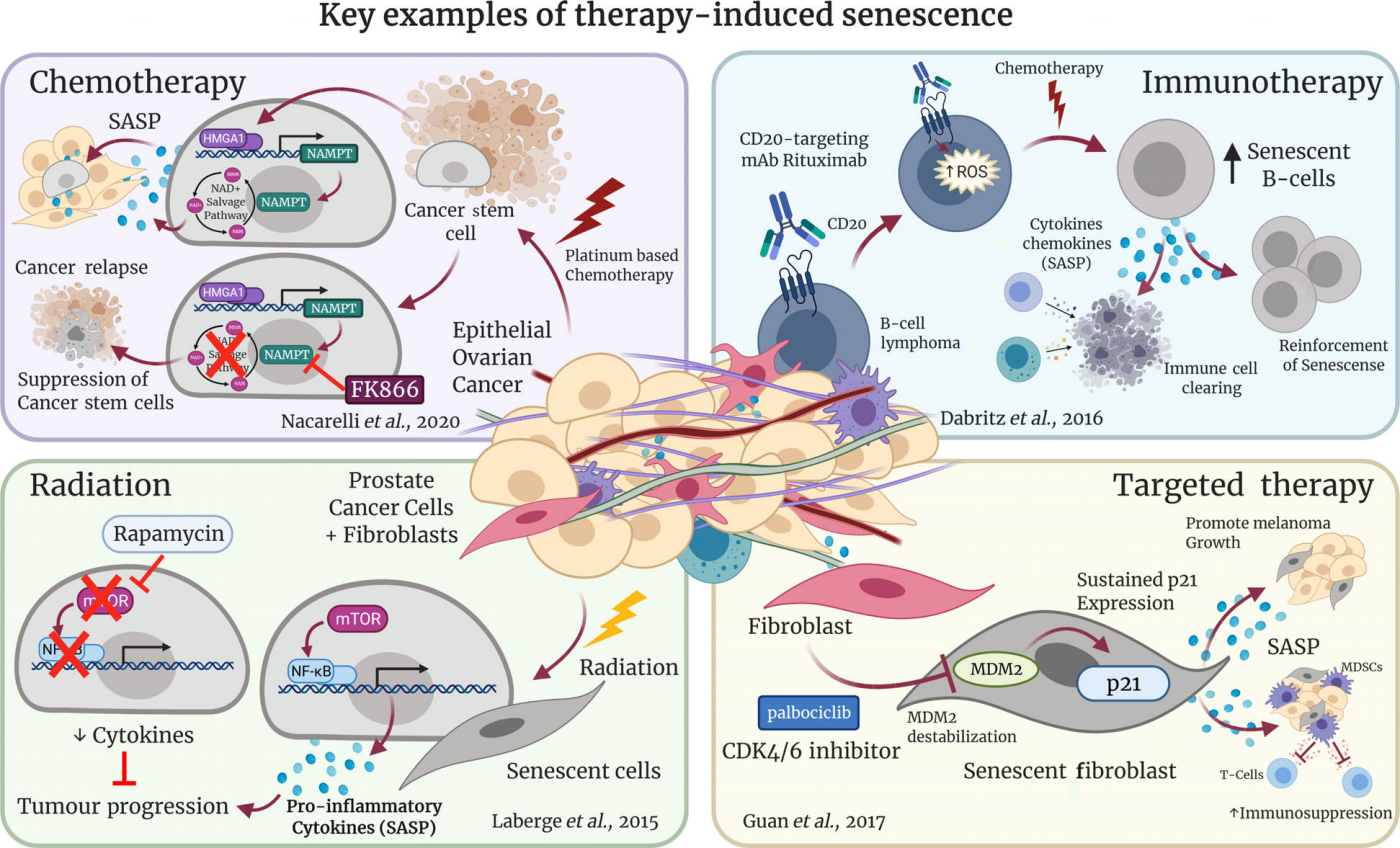
Key examples of therapy‐induced SASP. Many cancer therapy modalities induce senescence and associated SASP, and these can influence treatment efficacy in various ways. Chemotherapy (purple box): Nacarelli *et al*. (2020) showed that platinum‐based chemotherapy induces cellular senescence, which can promote the formation of cancer stem cells, eventually promoting tumour relapse [9]. Treatment with the NAMPT inhibitor FK866 suppressed these cancer stem cells, preventing outgrowth of cisplatin‐treated epithelial ovarian cancer cells. Radiation (green box): Laberge *et al*. (2015) showed that rapamycin (an mTOR inhibitor) prevented NFκB‐driven pro‐inflammatory SASP that is induced by radiation treatment of prostate cancer cells and fibroblasts, therefore inhibiting tumour progression [36]. Immunotherapy (blue box): Däbritz *et al*. (2016) used a CD20‐targeting monoclonal antibody to sensitise B‐cell lymphoma cells to senescence induction alongside chemotherapy [60]. This resulted in reinforcement of senescence and enhancement of immune cell action [60]. Targeted therapy (yellow box): Guan *et al*. (2017) showed that prolonged exposure to palbociclib, a CDK4/6 inhibitor (CDK4/6i), could induce senescence in normal fibroblasts in a DNA damage‐independent mechanism [53]. These fibroblasts exhibited MDM2 destabilisation and sustained p21^Cip1/Waf1^ expression, which resulted in the promotion of melanoma growth and enhanced immunosuppression [53].

Chemotherapy and radiation are common systemic modalities for the treatment of cancer and therefore can trigger the SASP in both cancer cells and surrounding TME cells [4]. Both treatments aim to cause catastrophic DNA damage, leading to a DDR, which can cause subsequent senescence and associated SASP. Logue *et al*. (2018), for example, reported that paclitaxel, a common chemotherapy for the treatment of triple‐negative breast cancer (TNBC), causes aberrant regulation of ER stress via enhanced inositol‐requiring enzyme 1 alpha (IRE1) RNAse activity [50]. This increase in IRE1 activity results in an increase in SASP‐associated factors such as IL‐6, IL‐8, CXCL1, granulocyte‐macrophage colony‐stimulating factor (GM‐CSF) and TGFβ2 [50]. When IRE1 RNAse activity was normalised, paclitaxel efficacy was enhanced in preclinical TNBC models, highlighting how the chemotherapy‐induced cancer cell secretome can drive tumour development and progression [50]. Furthermore, chemotherapy‐induced senescence and the resulting SASP can drive the expansion of cancer stem cells (CSCs) from dormant senescent cells, which are critical for cancer relapse. For example, Wang *et al*. (2019) showed that IL‐6 secretion induced by platinum treatment causes enrichment of CSCs in the residual tumours of high‐grade serous carcinoma (HGSC) [51]. This phenomenon was also shown by Nacarelli *et al*. (2020) [9], building on their previous study [46], which highlighted NAMPT as a key regulator of the SASP. In epithelial ovarian cancer (EOC), Nacarelli and colleagues showed that platinum‐induced senescence‐associated CSCs could be suppressed by treatment with the NAMPT inhibitor FK866 [9] (Fig. 2, see purple box). A combination of FK866 and cisplatin inhibited the outgrowth and eventual relapse of EOC, improving overall survival in these mice. Similarly, Shen *et al*. (2019) reported that breast cancer cells treated with docetaxel or Dox produce abundant EVs containing miRNAs that stimulate CSC expansion via targeting of ONECUT2, a master regulator of cell fate [52].

Incidental senescence due to systemic treatments can foster a SASP‐rich TME, which can not only potentiate tumorigenesis [5, 27, 53], but can also generate a multitude of side effects in normal tissues. These alterations in adjacent nontransformed tissue can cause reduced therapeutic efficacy in the short term [16, 54, 55], while also impacting quality‐of‐life (QoL) parameters in the long term [56]. For example, development of chronic inflammation via signalling molecules such as IL‐1A, IL‐6, IL‐8, CCL2 and CXCL is common in cancer patients, and consequences of systemic chemotherapy delivery are similar to those seen in ageing populations, such as frailty and insulin resistance [25]. As many of these cytokines also closely overlap with the SASP, it is possible that TIS is partly responsible for these adverse QoL side effects. Supporting this, Demaria *et al*. (2017) showed that treatment with a range of chemotherapy drugs, such as paclitaxel, Dox, temozolomide and cisplatin, induced a SASP in stromal host cells [18]. In this study, the authors utilised a transgenic mouse model (p16‐3MR) where host senescent cells can be tracked using a reporter gene under control of the senescence‐sensitive p16^INK4a^ promoter and additionally can also be eliminated by administering ganciclovir (GCV) [18]. They showed that chemotherapy‐induced senescence can promote cardiac dysfunction, bone marrow suppression, inflammation, frailty and fatigue, mirroring common side effects observed in human cancer patients when administered chemotherapy [18]. When these mice were orthotopically implanted with PyMT (polyoma middle tumour‐antigen) breast cancer cells and treated with Dox, those receiving the GCV dose to eliminate host senescent cells had significantly increased survival and decreased lung and liver metastases compared to Dox‐only controls, indicating that senescent host cells were pivotal to these protumorigenic processes [18]. Furthermore, Baar *et al*. (2017) showed that therapeutically targeting senescent cells in preclinical mouse models of Dox‐induced senescence can have the off‐target effect of improving liver function [57].

Strikingly, even after transcription and protein synthesis, anticancer treatments can influence SASP factor functionality. For example, the manipulation of the glycosylation state of secreted proteins has also been implicated in the chemotherapy‐induced secretome. In gastric cancer, Wu *et al*. (2016) identified that Dox‐ and vincristine‐resistant cancer cells secreted proteins which were uniquely glycosylated compared to parental lines using a glycosite enrichment mass spectrometry protocol [58]. These alterations in post‐translational modification add another layer of complexity to the characterisation of the chemotherapy‐induced secretome and could be used to better predict drug resistance in patients [58]. Other studies have also shown that reduction of senescent cells can potentially induce normalising effects on adjacent tissues and organs [57].

Although radiation therapy is generally a more targeted approach, it can still induce senescence in local adjacent cells, in addition to tumour cells. For example, Laberge *et al*. (2015) showed that multiple prostate and breast cancer lines, as well as fibroblasts, exhibit SASP after treatment with radiation [36] (Fig. 2, see green box). In this study, the authors showed that this was mTOR‐dependent, as treatment with rapamycin (an mTOR inhibitor) resulted in reduced cytokine levels, particularly those driven by NFκB signalling [36] (Fig. 2, see green box). Importantly, rapamycin inhibited prostate tumour growth *in vivo* via suppression of senescent fibroblasts (Fig. 2, see green box). Similarly, Sharma *et al*. (2016) reported that radiation exposure activates ADAM17 in non‐small cell lung cancer, resulting in the secretion of multiple SASP‐related factors, including ALCAM (activated leukocyte cell adhesion molecule) and amphiregulin, resulting in enhanced growth factor signalling and increased cytotoxicity in animal models [59].

Overall, having a more comprehensive understanding of SASP activation in chemotherapy‐ and radiation‐treated cancer cells and the TME will allow for the development of more tailored therapies, according to the patient’s response. If treatment can be precisely regulated to benefit not just survival, but also the patient’s treatment experience by reducing some of the extreme side effects as discussed, it will be beneficial for a wide‐reaching population of cancer patients.

### 2.2 The immunomodulatory roles of SASP in the context of immunotherapy

As immunotherapy is a relatively new modality in cancer treatment, few studies have investigated how senescence, and the associated SASP, is implicated in its therapeutic mechanisms. Nevertheless, there are a few indications that immunotherapy can drive TIS. For example, Däbritz *et al*. (2016) reported that rituximab, a CD20‐targeting monoclonal antibody, elevated intracellular reactive oxygen species (ROS) in B‐cell lymphoma, inducing and enhancing a SASP programme in these cells [60] (Fig. 2, see blue box). In this study, SASP was shown to sensitise these cells to the chemotherapy drugs Dox and vincristine, resulting in a pro‐inflammatory network that not only reinforced senescent behaviour, but also improved immune cell clearing [60] (Fig. 2, see blue box).

As it stands, it is still unclear how prominent the SASP phenomenon is following immunotherapy as a stand‐alone treatment, with further research required. The majority of evidence in this area has focussed on targeting senescent cells induced by first‐line treatments, to then enhance the efficacy of immunotherapy. For example, Zhao *et al*. (2020) revealed that ovarian cancer cells exposed to cisplatin underwent a SASP that was dependent on topoisomerase 1‐DNA covalent cleavage complex (TOP1cc). Knockdown of HMGB2, which stabilises TOP1cc, resulted in decreased efficacy of anti‐PD‐1 blockade, suggesting that this signalling axis plays a key role in checkpoint efficacy [61]. Hao *et al*. (2020) then built upon these results by combining cisplatin with a topoisomerase 1 (TOP1) inhibitor, which promotes the formation of TOP1cc by sequestering the complex on the DNA. This combination therapy boosted the SASP, thereby sensitising the ovarian cells to anti‐PD‐1 treatment, which resulted in increased infiltration of activated T cells and dendritic cells (DCs) [62]. Furthermore, Jerby‐Arnon and colleagues (2018) used single‐cell RNA sequencing (sc‐RNAseq) to identify an anti‐PD‐1‐resistant population in melanoma [63]. From here, they found that CDK4/6 inhibition reversed this resistant phenotype by inducing components of SASP, therefore enhancing the effectiveness of checkpoint blockade [63].

It is important to note that, although SASP can be immunostimulatory in some cellular contexts, there are also many settings where SASP can instead drive an immunosuppressive phenotype. For example, in Pten‐null prostate tumours, activation of the JAK2/STAT3 signalling pathway promotes a senescent phenotype where secretion of pro‐inflammatory cytokines such as CXCL1/CXCL2, IL‐6 and macrophage colony‐stimulating factor (M‐CSF) recruits myeloid‐derived suppressor cells to the tumour site, thereby inhibiting effective cytotoxic T‐cell responses [37]. Combination of a JAK2 inhibitor with chemotherapy inhibited this SASP pro‐inflammatory network, increasing infiltration of T cells and decreasing tumour burden [37]. Interestingly, the immunosuppressive effects of the SASP are not only localised to the primary tumour. For example, Luo *et al*. (2016) reported that senescent osteoblasts secreting IL‐6 increased local bone resorption, thereby fostering a prometastatic niche in the bone of breast cancer patients, driving metastatic outgrowth [64]. This group later found that the IL‐6 secreted by senescent stromal cells can recruit myeloid‐derived suppressor cells, inhibiting effective antitumour T‐cell responses [14]. It is clear that, in settings where SASP induces an immunosuppressive phenotype, ablation of these senescent cells could help stimulate antitumour immune responses. Recently, chimeric antigen receptor (CAR) T cells against a broad senescent cell marker, urokinase‐type plasminogen activator receptor (uPAR), have been utilised to ablate senescent cells during liver fibrosis and lung adenocarcinomas, exhibiting increased survival and decreased associated toxicities, and have exciting implications for the ablation of protumorigenic senescent cells in other cancer types [65].

Cancer cell‐derived lysosomes, although not strictly a SASP component, can also have an immunomodulatory effect in the TME, reducing efficacy of immunotherapy in melanoma. This was shown by Satana‐Magal *et al*. (2020): tumour‐infiltrating DCs, which usually enhance cytotoxic T‐cell‐mediated cancer killing, were progressively depleted in mouse models of melanoma [66]. The authors established that the DCs were undergoing apoptosis due to their uptake of cancer cell lysosomes, which resulted in incomplete clearance of melanoma lesions and reduced immunotherapy efficacy [66]. This study highlights the diversity of cancer cell secretions in the context of therapy resistance for emerging anticancer therapies such as immunotherapy. As the SASP plays a fundamental immunomodulatory role in many cancer types, further understanding and manipulation of this phenotype might represent an important step towards enhancing the efficacy of immunotherapy and could also help explain the variation in patient response often found in the clinical setting.

### 2.3 Targeted cancer therapies can induce a detrimental SASP

Targeted kinase inhibitors for specific oncogenic drivers are an emerging area within oncology and have been shown to be clinically effective in some settings; however, complete tumour remission is rare due to development of chemoresistance or adverse effects [67]. For example, hyperactivity of the cyclin‐dependent kinase proteins (CDKs) is often observed in cancer, as it allows the cells to pass through the cell cycle at an accelerated rate [68]. In the past two decades, CDK4/6 inhibitors (CDK4/6i) have offered promising opportunities for normalising the continual proliferative capabilities of malignant cells. By initiating cell cycle arrest at the G1 phase, CDK4/6i have been shown to effectively block the normal function of CDK4 and CDK6, thereby halting the transition into S phase, which allows the cell to cycle [69]. As their primary goal is to halt the cell cycle, CDK4/6i can induce a transient senescence of cancer cells [70], and therefore, their ability to produce a damaging SASP must be considered. Stemming from their original work in 2009 [32], Guan *et al*. (2017) recently showed that prolonged exposure to palbociclib, a CDK4/6i, could induce senescence in normal fibroblasts in a DNA damage‐independent mechanism [53] (Fig. 2, see yellow box). They showed that senescent fibroblasts harboured an increased degradation of Mdm2, promoting downregulation of p53, while also holding elevated p21^Cip1/Waf1^ expression, the cumulation of which they suggest triggers senescence and activates the pro‐inflammatory SASP (Fig. 2, see yellow box). *In vivo*, the senescent fibroblasts aided tumorigenesis in four genetically distinct lines of melanoma [53]. Although the authors referred to this DNA damage‐independent mechanism in a previous publication [71], further work should be considered regarding this noncanonical senescence pathway to confirm that the protumorigenic effects of the CDK4/6i‐induced SASP are truly causative of this transcriptional signature activity.

More recently, Wang *et al*. (2020) have shown that abemaciclib and palbociclib did indeed induce senescence in resident fibroblasts but did not induce the NFκB‐mediated and pro‐inflammatory SASP [72]. This again was the result of a DNA damage‐independent mechanism, and the authors showed that the SASP from some of the senescent fibroblasts lacked the normal pro‐inflammatory phenotype and instead was driven by p53 transcriptional activity instead of NFκB, which produced an independent programme that the authors termed ‘PASP’ [72]. The PASP‐only expressing cells, in this circumstance, were reported to be cleared more quickly by the immune system than the NFκB‐driven SASP cells (named ‘NASP’), thereby removing the possibility of detrimental effects of senesced nonmalignant cells, reported in previous studies [53]. This study may challenge the previously established dogma that senescence of nonmalignant cells always contributes to enhanced tumorigenesis and also brings to light that distinct phenotypes of the SASP can be driven by differing transcriptional programmes.

Other kinase inhibitors have also been implicated in inducing a detrimental SASP. For example, Obenauf *et al*. (2015) found that treatment with kinase inhibitors against BRAF, ALK (anaplastic lymphoma kinase) or EGFR mutations significantly modulates the secretome of drug‐sensitive cancer cells. This drug‐altered secretome paradoxically creates a tumour microenvironment which supports inhibitor‐resistant cancer cell clones, promoting tumour progression [54]. Interestingly, Mastri *et al*. (2018) reported that a VEGFR tyrosine kinase inhibitor can induce a ‘transient pseudosenescent secretory’ phenotype in cancer cells, which have become resistant to the treatment. Once treatment is halted, these cells drive significant rebound growth at both primary and metastatic sites in preclinical models [73]. Similarly, Wang *et al*. (2019) reported that fibroblast growth factor receptor (FGFR) inhibitor resistance in lung cancer is initially mediated by the cancer cell secretome activating STAT3 signalling; however, they also showed that this resistance could be enhanced via interactions with both fibroblasts and macrophages [74]. Rho‐associated protein kinase (ROCK) inhibitors have recently been reported to improve chemotherapy delivery and reduce metastases in cancers such as pancreas and breast [75, 76, 77]. Interestingly, Niklander *et al*. (2020) found that the ROCK inhibitor Y‐27632 could decrease activity of several factors within the pro‐inflammatory SASP, without altering cell cycle arrest in oral dysplastic keratinocytes [78]. The authors show that this is mediated by IL‐1 inhibition from Y‐27632, which in turn regulated the secretion of protumorigenic SASP components such as IL‐6 and IL‐8 [78]. This study therefore offers an interesting insight into, and highlights a potential added benefit of, ROCK inhibition: the reduced production of pro‐inflammatory and protumorigenic IL‐6 and IL‐8.

On the whole, kinase therapies offer new hope for improving responses in specific cancers by tailoring the antitumorigenic effects to known molecular mechanisms occurring within that cancer. Less is known about the consequences of kinase‐therapy‐induced senescence and associated SASP, and more work will be required to understand the apparent cancer type‐ and therapy‐driven differences.

## 3 Senolytics and senostatics: targeting and utilising the SASP

Considering the potential risk of accumulating protumorigenic senescent cells, novel mechanisms to selectively target senescent cancer cells following treatment are now being researched. Moreover, the concept of synthetic lethality [79], also known as the ‘two‐step’ approach, has gained traction in recent times, with growing evidence exploring the idea of inducing vulnerability in cancer cells to then eliminate them with a second agent [80]. Following TIS, second‐line therapies can target senescent cells by two methods: (a) selectively killing the senescent cells and therefore removing the possibility of the SASP impacting tumorigenesis; or (b) directly targeting and inhibiting the SASP‐related factors, thereby reducing the potential for toxic reactions in the TME [81, 82]. These exciting therapeutic approaches, known under the broad terms of senolytics and senostatics, respectively, will be discussed further below (and summarised in Table 1).

**Table 1:** Summary table of senolytic and senostatic drugs currently being utilised to target SASP in various cancers. BCL‐2, B‐cell lymphoma 2; EOC, epithelial ovarian cancer; FBP1, fructose‐bisphosphatase 1; HCC, hepatocellular carcinoma; HNSCC, head and neck squamous cell carcinoma; NAMPT, nicotinamide phosphoribosyltransferase; PARP, poly(ADP‐ribose) polymerase; TNBC, triple‐negative breast cancer; TOP1, topoisomerase 1; uPAR, urokinase‐type plasminogen activator receptor.

Drug name	Target	Effect	References
Senolytics			
ABT‐263 (Navitoclax)	BLC‐2 inhibitor	↓ HCC tumour growth following FBP1 loss via SASP inhibition	[44, 85, 86]
		↓ PARP inhibitor induced senescent cells in ovarian and breast cancer	
		↓ Metastatic burden by targeting *TIMP1*‐deficient senescent cells in prostate cancer	
GX15‐070 (Obatoclax)	BCL‐2 inhibitor	↑ Apoptosis of senescent cells induced by BET inhibition in TNBC	[84, 87]
		↓ Senescent cells induced by BMI1 inhibition enhancing tumour killing in DIPG	
uPAR CAR T cells	uPAR	↓ Senescent cells extending lung adenocarcinoma survival.	[65]
Senostatics			
Dasatinib	SRC‐family protein‐tyrosine kinase inhibitor	↓ HCC tumour growth following FBP1 loss via SASP inhibition	[44]
FK866/GMX1778	NAMPT inhibitor	↓ Senescent‐associated cancer stem cell outgrowth in EOC	[9, 46]
Irinotecan	TOP1 inhibitor	↑ SASP in ovarian cancer sensitising cells to anti‐PD‐1 therapy	[62]
Metformin	Gluconeogenesis inhibition	↓ mTOR and STAT3 pathway signalling in response to a CDK4/6 inhibitor repressing the stemness of HNSCC cancer cells.	[89]
NVP‐BSK805	JAK2 inhibitor	↓ SASP in *Pten*‐deficient prostate tumours leading to increased CD8 activity	[36]
Rapamycin	mTOR inhibitor	↓ Senescent fibroblasts in prostate cancer inhibiting tumour growth	[35]
Sertraline	Serotonin reuptake inhibitor	↓ mTOR signalling in HCC senescent cells causing apoptosis and reduction of tumour growth	[90]
Y‐27632	ROCK inhibitor	↓ IL‐6 and IL‐8 production from senescent oral keratinocytes	[78]

Senescent cells are able to persist without cell death, partly due to an upregulation of anti‐apoptotic network pathways [83], and as such, many senolytic studies involve targeting the B‐cell lymphoma 2 (BCL‐2) family, which regulates cell apoptosis to enhance senescent cancer cell killing. For example, GX15‐070 (obatoclax mesylate), a BCL‐2 inhibitor, was shown to improve bromodomain and extraterminal protein inhibitors (BETi) in TNBC [84]. Similarly, another BCL‐2 inhibitor, ABT‐263 (Navitoclax), was reported to eliminate senescent cells and enhance the efficacy of olaparib, a poly(ADP‐ribose) polymerase (PARP) inhibitor, in both breast and ovarian cancer cell lines [85]. Furthermore, in a preclinical model of prostate cancer, *TIMP1* loss, a pan‐matrix metalloproteinase inhibitor, was reported to promote metastasis in senescent tumours [86]. When targeting *TIMP1*‐deficient senescent tumour cells with ABT‐263, the authors showed that the metastatic potential was significantly reduced. Moreover, docetaxel treatment, which is standard chemotherapy for prostate cancer, perpetrated TIS and exacerbated metastasis in *TIMP1*‐deficient cancer cells, but this reaction could be rescued with ABT‐263, thereby reducing metastatic events [86].

Balakrishnan *et al*. (2020) have recently used obatoclax, a BH3 mimetic, in diffuse intrinsic pontine glioma (DIPG), an aggressive paediatric brain tumour. Due to an epigenomic mutation of H3K27M that is characteristic of the disease, increased expression of a polycomb complex protein, BMI1, causes cancer cells to senesce and drive the SASP [87]. By inhibiting BMI1 using a small molecule inhibitor and combining this with obatoclax, the authors showed significant improvement in survival and tumour regression [87]. Delay to tumour regrowth was sustained after drugs were removed, suggesting that this dual therapy could have long‐term benefits. This study highlights an exciting senolytic therapy option for a normally fatal childhood brain tumour. This preclinical work also supports an ongoing clinical trial in which a BMI1 small molecule inhibitor (PTC596) is being used in combination with radiotherapy, which was started without substantial *in vivo* research (NCT03605550). Similarly, Ruscetti *et al*. (2020) showed that treatment with CDK4/6i triggered the production of pro‐angiogenic SASP factors such as VEGF, basic FGF (bFGF), PDGFα and β, as well as multiple MMPs, resulting in vascular remodelling in preclinical models of PDAC [20]. In this study, the authors showed that this enhanced gemcitabine chemotherapy delivery to the tumours, while also enhancing cytotoxic T‐cell infiltration [20]. Then, due to this improved immunogenicity, the efficacy of checkpoint blockade via anti‐PD‐1 was enhanced, increasing overall survival [20]. However, although this study provides strong rationale for combining SASP‐inducing therapies with other treatment modalities, such as chemotherapy and/or immunotherapy, layering treatments in this manner will likely increase the risk of toxicity in cancer patients, with further research required to elucidate whether this is a viable therapeutic route within the clinic.

It is important to remember that, although senescence does play a role in these detrimental effects after cancer therapy, it has also evolved to accompany humans as they age. Recent work has shown that senescent cells may remain biologically functional in some situations, such as in pancreatic β‐cells, where senescent cells increase secretion of insulin to compensate for decreased proliferation [88]. Therefore, the use of senolytic therapy should be considered with caution, and much more research is needed prior to application in humans.

A more moderate approach may be instead to modulate factors secreted by senescent cells through the use of senostatics. However, as previously discussed, the SASP shares many overlapping secreted factors that have important physiological functions. As such, many researchers are now working to identify a more precise SASP. For example, in a large‐scale small interfering RNA (siRNA) screen for regulators of SASP, Georgilis *et al*. (2018) identified 50 genes, including splicing factor PTBP1, whose depletion prevents the protumorigenic effects of SASP, such as NFκB‐driven inflammation, without impacting senescence growth arrest [19]. This study highlights that targeting specific aspects of SASP may be the key to exploiting this complex reaction, especially when the malignancy is driven by inflammation [19].

Regulating the activity of the SASP has also been shown to be effective in head and neck squamous cell carcinoma (HNSCC) by repurposing the hyperglycaemic drug metformin [89]. In this study, Hu *et al*. (2020) demonstrate that the efficacy of a CDK4/6i (LY2835219) can be potentiated by administering it in combination with metformin. LY2835219 successfully prevented cell cycling into S phase, whereas metformin inhibited mTOR and STAT3 pathways that are normally upregulated in the SASP by senescent HNSCC cells following CDK4/6 inhibition [89]. Furthermore, the synergistic effect of combining the drugs resulted in a more efficient cell cycle arrest than when LY2835219 was administered alone and, when combined, reduced the expression of several pro‐inflammatory proteins known to be within the SASP and to aid tumorigenesis, such as IL‐6, IL‐8 and CXCL2 [89]. Overall, the authors showed that, by regulating the SASP following CDK4/6 inhibition, the stemness of the cancer cells could be repressed via the IL‐6‐STAT3 axis. Similarly, Wang *et al*. (2019) reported that targeting liver cancer cells with CDC7, a DNA‐replication kinase, followed by sertraline, a commonly prescribed antidepressant, enhanced cancer cell death. In this study, targeting CDC7 induced senescence and the associated SASP, after which sertraline triggered apoptosis via suppressed mTOR signalling [90]. These studies are exciting examples of a growing field where FDA‐approved drugs are repurposed in other disease settings. Considering the long journey to developing FDA‐approved drugs, this offers new hope for a fast‐tracked approach to cancer therapy.

## 4 Future perspectives

Many chemotherapeutic agents are successful in tackling cancer by inflicting high levels of DNA damage, but only recently have we begun to understand the secondary and often opposing effects of these treatments. Senescence and the associated SASP can affect not only malignant cells, but also cells within the TME, which have been shown to be detrimental in several preclinical models of cancer. Aside from assisting in tumorigenesis, the SASP has also been shown to affect other unwanted side effects of chemotherapies. For example, Dox‐treated fibroblasts became senescent and secreted SASP factors associated with haemostasis, where activated platelets produced harmful blood clots [91]. Furthermore, evidence from the past decade is collectively highlighting that the SASP is a particularly diverse phenomenon that can be manipulated depending on the stressor‐derived transcriptional driver and the cell that it is occurring in. In particular, studies have recently shown that there are highly pro‐inflammatory SASPs that promote the secretion of protumorigenic factors such as IL‐1A and IL‐6 and less inflammatory SASPs that promote the clearance of senescent cells by the immune system, by secreting factors such as TGF‐β [31]. Novel investigations are now pursuing the idea of either clearing senescent and SASP‐producing cells or equilibrating the activity of specific SASP factors by pharmacological inhibition in combination with primary anticancer therapies. This remains a difficult but important area of research, as we are still learning of the protective elements of the SASP after TIS and eliminating all SASP‐related factors might impact other physiological functions, such as wound healing [92]. Overall, the research of senotherapies is an extremely promising area of cancer pharmacology that may in the future reduce the adverse effects of senescence and the SASP following cancer therapy.

## Conflict of interest

The authors declare no conflict of interest.

## References

[1] Campisi J & d'Adda di Fagagna F (2007) Cellular senescence: when bad things happen to good cells. Nat Rev Mol Cell Biol 8, 729–740. 17667954 10.1038/nrm2233

[2] Martincorena I , Raine KM , Gerstung M , Dawson KJ , Haase K , Van Loo P , Davies H , Stratton MR & Campbell PJ (2017) Universal patterns of selection in cancer and somatic tissues. Cell 171, 1029–1041. 10.1016/j.cell.2017.09.042 29056346 PMC5720395

[3] Hernandez‐Segura A , Nehme J & Demaria M (2018) Hallmarks of cellular senescence. Trends Cell Biol 28, 436–453. 29477613 10.1016/j.tcb.2018.02.001

[4] Wang B , Kohli J & Demaria M (2020) Senescent cells in cancer therapy: friends or foes? Trends Cancer 6, 838–857. 32482536 10.1016/j.trecan.2020.05.004

[5] Coppé J‐P , Patil CK , Rodier F , Sun Y , Muñoz DP , Goldstein J , Nelson PS , Desprez P‐Y & Campisi J (2008) Senescence‐associated secretory phenotypes reveal cell‐nonautonomous functions of oncogenic RAS and the p53 tumor suppressor. PLoS Biol 6, e301. 10.1371/journal.pbio.0060301 PMC2592359 19053174

[6] Canino C , Mori F , Cambria A , Diamantini A , Germoni S , Alessandrini G , Borsellino G , Galati R , Battistini L , Blandino R *et al*., (2012) SASP mediates chemoresistance and tumor‐initiating‐activity of mesothelioma cells. Oncogene 31, 3148–3163. 22020330 10.1038/onc.2011.485

[7] Laberge RM , Awad P , Campisi J & Desprez PY (2012) Epithelial‐mesenchymal transition induced by senescent fibroblasts. Cancer Microenviron 5, 39–44. 21706180 10.1007/s12307-011-0069-4 PMC3343197

[8] Milanovic M , Fan DNY , Belenki D , Däbritz JHM , Zhao Z , Yu Y , Dörr JR , Dimitrova L , Lenze D , Monteiro Barbosa IA *et al*., (2018) Senescence‐associated reprogramming promotes cancer stemness. Nature 553, 96–100. 29258294 10.1038/nature25167

[9] Nacarelli T , Fukumoto T , Zundell JA , Fatkhutdinov N , Jean S , Cadungog MG , Borowsky ME & Zhang R (2020) NAMPT inhibition suppresses cancer stem‐like cells associated with therapy‐induced senescence in ovarian cancer. Cancer Res 80, 890–900. 31857293 10.1158/0008-5472.CAN-19-2830 PMC7024650

[10] Kim YH , Choi YW , Lee J , Soh EY , Kim J‐H & Park TJ (2017) Senescent tumor cells lead the collective invasion in thyroid cancer. Nat Commun 8, 1–14. 28489070 10.1038/ncomms15208 PMC5436223

[11] Coppé J‐P , Kauser K , Campisi J & Beauséjour CM (2006) Secretion of vascular endothelial growth factor by primary human fibroblasts at senescence. J Biol Chem 281, 29568–29574. 16880208 10.1074/jbc.M603307200

[12] Toste PA , Nguyen AH , Kadera BE , Duong M , Wu N , Gawlas I , Tran LM , Bikhchandani M , Li L , Patel SG *et al*., (2016) Chemotherapy‐induced inflammatory gene signature and protumorigenic phenotype in pancreatic CAFs via stress‐associated MAPK. Mol Cancer Res 14, 437–447. 26979711 10.1158/1541-7786.MCR-15-0348 PMC4867256

[13] Eggert T , Wolter K , Ji J , Ma C , Yevsa T , Klotz S , Medina‐Echeverz J , Longerich T , Forgues M , Reisinger F *et al*., (2016) Distinct functions of senescence‐associated immune responses in liver tumor surveillance and tumor progression. Cancer Cell 30, 533–547. 27728804 10.1016/j.ccell.2016.09.003 PMC7789819

[14] Ruhland MK , Loza AJ , Capietto A‐H , Luo X , Knolhoff BL , Flanagan KC , Belt BA , Alspach E , Leahy K , Luo J *et al*., (2016) Stromal senescence establishes an immunosuppressive microenvironment that drives tumorigenesis. Nat Commun 7, 11762. 27272654 10.1038/ncomms11762 PMC4899869

[15] Angelini PD , Zacarias Fluck MF , Pedersen K , Parra‐Palau JL , Guiu M , Bernadó Morales C , Vicario R , Luque‐García A , Navalpotro NP , Giralt J *et al*., (2013) Constitutive HER2 signaling promotes breast cancer metastasis through cellular senescence. Cancer Res 73, 450–458. 23288917 10.1158/0008-5472.CAN-12-2301

[16] Demaria M , O'Leary MN , Chang J , Shao L , Liu S , Alimirah F , Koenig K , Le C , Mitin N & Deal AM (2017) Cellular senescence promotes adverse effects of chemotherapy and cancer relapse. Cancer Discov 7, 165–176. 27979832 10.1158/2159-8290.CD-16-0241 PMC5296251

[17] Georgilis A , Klotz S , Hanley CJ , Herranz N , Weirich B , Morancho B , Leote AC , D'Artista L , Gallage S , Seehawer M *et al*., (2018) PTBP1‐mediated alternative splicing regulates the inflammatory secretome and the pro‐tumorigenic effects of senescent cells. Cancer Cell 34, 85–102.e109. 29990503 10.1016/j.ccell.2018.06.007 PMC6048363

[18] Jackson JG , Pant V , Li Q , Chang LL , Quintás‐Cardama A , Garza D , Tavana O , Yang P , Manshouri T , Li Y *et al*., (2012) p53‐mediated senescence impairs the apoptotic response to chemotherapy and clinical outcome in breast cancer. Cancer Cell 21, 793–806. 22698404 10.1016/j.ccr.2012.04.027 PMC3376352

[19] Sun Y , Campisi J , Higano C , Beer TM , Porter P , Coleman I , True L & Nelson PS (2012) Treatment‐induced damage to the tumor microenvironment promotes prostate cancer therapy resistance through WNT16B. Nat Med 18, 1359–1368. 22863786 10.1038/nm.2890 PMC3677971

[20] Ruscetti M , Morris JP , Mezzadra R , Russell J , Leibold J , Romesser PB , Simon J , Kulick A , Ho Y‐J , Fennell M *et al*., (2020) Senescence‐induced vascular remodeling creates therapeutic vulnerabilities in pancreas cancer. Cell 181, 424–441.e421. 32234521 10.1016/j.cell.2020.03.008 PMC7278897

[21] Chien Y , Scuoppo C , Wang X , Fang X , Balgley B , Bolden JE , Premsrirut P , Luo W , Chicas A & Lee CS (2011) Control of the senescence‐associated secretory phenotype by NF‐κB promotes senescence and enhances chemosensitivity. Genes Dev 25, 2125–2136. 21979375 10.1101/gad.17276711 PMC3205583

[22] Iannello A , Thompson TW , Ardolino M , Lowe SW & Raulet DH (2013) p53‐dependent chemokine production by senescent tumor cells supports NKG2D‐dependent tumor elimination by natural killer cells. J Exp Med 210, 2057–2069. 24043758 10.1084/jem.20130783 PMC3782044

[23] Kang T‐W , Yevsa T , Woller N , Hoenicke L , Wuestefeld T , Dauch D , Hohmeyer A , Gereke M , Rudalska R & Potapova A (2011) Senescence surveillance of pre‐malignant hepatocytes limits liver cancer development. Nature 479, 547–551. 22080947 10.1038/nature10599

[24] Cupit‐Link MC , Kirkland JL , Ness KK , Armstrong GT , Tchkonia T , LeBrasseur NK , Armenian SH , Ruddy KJ & Hashmi SK (2017) Biology of premature ageing in survivors of cancer. ESMO Open 2, e000250. 29326844 10.1136/esmoopen-2017-000250 PMC5757468

[25] Acosta JC , O'Loghlen A , Banito A , Guijarro MV , Augert A , Raguz S , Fumagalli M , Da Costa M , Brown C , Popov N *et al*., (2008) Chemokine signaling via the CXCR2 receptor reinforces senescence. Cell 133, 1006–1018. 18555777 10.1016/j.cell.2008.03.038

[26] Kuilman T , Michaloglou C , Vredeveld LC , Douma S , van Doorn R , Desmet CJ , Aarden LA , Mooi WJ & Peeper DS (2008) Oncogene‐induced senescence relayed by an interleukin‐dependent inflammatory network. Cell 133, 1019–1031. 18555778 10.1016/j.cell.2008.03.039

[27] Acosta JC , Banito A , Wuestefeld T , Georgilis A , Janich P , Morton JP , Athineos D , Kang TW , Lasitschka F , Andrulis M *et al*., (2013) A complex secretory program orchestrated by the inflammasome controls paracrine senescence. Nat Cell Biol 15, 978–990. 23770676 10.1038/ncb2784 PMC3732483

[28] Takasugi M , Okada R , Takahashi A , Virya Chen D , Watanabe S & Hara E (2017) Small extracellular vesicles secreted from senescent cells promote cancer cell proliferation through EphA2. Nat Commun 8, 15729. 10.1038/ncomms15728 PMC5467215 28585531

[29] Campisi J (2013) Aging, cellular senescence, and cancer. Annu Rev Physiol 75, 685–705. 23140366 10.1146/annurev-physiol-030212-183653 PMC4166529

[30] Faget DV , Ren Q & Stewart SA (2019) Unmasking senescence: context‐dependent effects of SASP in cancer. Nat Rev Cancer 19, 439–453. 31235879 10.1038/s41568-019-0156-2

[31] Rodier F , Coppé J‐P , Patil CK , Hoeijmakers WA , Muñoz DP , Raza SR , Freund A , Campeau E , Davalos AR & Campisi J (2009) Persistent DNA damage signalling triggers senescence‐associated inflammatory cytokine secretion. Nat Cell Biol 11, 973–979. 19597488 10.1038/ncb1909 PMC2743561

[32] Goehe RW , Di X , Sharma K , Bristol ML , Henderson SC , Valerie K , Rodier F , Davalos AR & Gewirtz DA (2012) The autophagy‐senescence connection in chemotherapy: must tumor cells (self) eat before they sleep? J Pharmacol Exp Ther 343, 763–778. 22927544 10.1124/jpet.112.197590 PMC3500537

[33] Alspach E , Flanagan KC , Luo X , Ruhland MK , Huang H , Pazolli E , Donlin MJ , Marsh T , Piwnica‐Worms D , Monahan J *et al*., (2014) p38MAPK plays a crucial role in stromal‐mediated tumorigenesis. Cancer Discov 4, 716–729. 24670723 10.1158/2159-8290.CD-13-0743 PMC4049323

[34] Freund A , Patil CK & Campisi J (2011) p38MAPK is a novel DNA damage response‐independent regulator of the senescence‐associated secretory phenotype. EMBO J 30, 1536–1548. 21399611 10.1038/emboj.2011.69 PMC3102277

[35] Laberge R‐M , Sun Y , Orjalo AV , Patil CK , Freund A , Zhou L , Curran SC , Davalos AR , Wilson‐Edell KA & Liu S (2015) MTOR regulates the pro‐tumorigenic senescence‐associated secretory phenotype by promoting IL1A translation. Nat Cell Biol 17, 1049–1061. 26147250 10.1038/ncb3195 PMC4691706

[36] Toso A , Revandkar A , Di Mitri D , Guccini I , Proietti M , Sarti M , Pinton S , Zhang J , Kalathur M , Civenni G *et al*., (2014) Enhancing chemotherapy efficacy in Pten‐deficient prostate tumors by activating the senescence‐associated antitumor immunity. Cell Rep 9, 75–89. 25263564 10.1016/j.celrep.2014.08.044

[37] Coppe JP , Rodier F , Patil CK , Freund A , Desprez PY & Campisi J (2011) Tumor suppressor and aging biomarker p16(INK4a) induces cellular senescence without the associated inflammatory secretory phenotype. J Biol Chem 286, 36396–36403. 21880712 10.1074/jbc.M111.257071 PMC3196093

[38] Efeyan A , Ortega‐Molina A , Velasco‐Miguel S , Herranz D , Vassilev LT & Serrano M (2007) Induction of p53‐dependent senescence by the MDM2 antagonist nutlin‐3a in mouse cells of fibroblast origin. Cancer Res 67, 7350–7357. 17671205 10.1158/0008-5472.CAN-07-0200

[39] Wiley CD , Schaum N , Alimirah F , Lopez‐Dominguez JA , Orjalo AV , Scott G , Desprez PY , Benz C , Davalos AR & Campisi J (2018) Small‐molecule MDM2 antagonists attenuate the senescence‐associated secretory phenotype. Sci Rep 8, 2410. 29402901 10.1038/s41598-018-20000-4 PMC5799282

[40] Hernandez‐Segura A , de Jong TV , Melov S , Guryev V , Campisi J & Demaria M (2017) Unmasking transcriptional heterogeneity in senescent cells. Curr Biol 27, 2652–2660.e4. 28844647 10.1016/j.cub.2017.07.033 PMC5788810

[41] Debacq‐Chainiaux F , Erusalimsky JD , Campisi J & Toussaint O (2009) Protocols to detect senescence‐associated beta‐galactosidase (SA‐betagal) activity, a biomarker of senescent cells in culture and in vivo. Nat Protoc 4, 1798–1806. 20010931 10.1038/nprot.2009.191

[42] Basisty N , Kale A , Jeon OH , Kuehnemann C , Payne T , Rao C , Holtz A , Shah S , Sharma V , Ferrucci L *et al*., (2020) A proteomic atlas of senescence‐associated secretomes for aging biomarker development. PLoS Biol 18, e3000599. 31945054 10.1371/journal.pbio.3000599 PMC6964821

[43] Yoshimoto S , Loo TM , Atarashi K , Kanda H , Sato S , Oyadomari S , Iwakura Y , Oshima K , Morita H , Hattori M *et al*., (2013) Obesity‐induced gut microbial metabolite promotes liver cancer through senescence secretome. Nature 499, 97–101. 23803760 10.1038/nature12347

[44] Li F , Huangyang P , Burrows M , Guo K , Riscal R , Godfrey J , Lee KE , Lin N , Lee P , Blair IA *et al*., (2020) FBP1 loss disrupts liver metabolism and promotes tumorigenesis through a hepatic stellate cell senescence secretome. Nat Cell Biol 22, 728–739. 32367049 10.1038/s41556-020-0511-2 PMC7286794

[45] Dorr JR , Yu Y , Milanovic M , Beuster G , Zasada C , Dabritz JH , Lisec J , Lenze D , Gerhardt A , Schleicher K *et al*., (2013) Synthetic lethal metabolic targeting of cellular senescence in cancer therapy. Nature 501, 421–425. 23945590 10.1038/nature12437

[46] Nacarelli T , Lau L , Fukumoto T , Zundell J , Fatkhutdinov N , Wu S , Aird KM , Iwasaki O , Kossenkov AV , Schultz D *et al*., (2019) NAD(+) metabolism governs the proinflammatory senescence‐associated secretome. Nat Cell Biol 21, 397–407. 30778219 10.1038/s41556-019-0287-4 PMC6448588

[47] Galli U , Colombo G , Travelli C , Tron GC , Genazzani AA & Grolla AA (2020) Recent advances in NAMPT inhibitors: a novel immunotherapic strategy. Front Pharmacol 11, 656. 32477131 10.3389/fphar.2020.00656 PMC7235340

[48] Heske CM (2019) Beyond energy metabolism: exploiting the additional roles of NAMPT for cancer therapy. Front Oncol 9, 1514. 32010616 10.3389/fonc.2019.01514 PMC6978772

[49] Tonnessen‐Murray CA , Frey WD , Rao SG , Shahbandi A , Ungerleider NA , Olayiwola JO , Murray LB , Vinson BT , Chrisey DB , Lord CJ *et al*., (2019) Chemotherapy‐induced senescent cancer cells engulf other cells to enhance their survival. J Cell Biol 218, 3827–3844. 31530580 10.1083/jcb.201904051 PMC6829672

[50] Logue SE , McGrath EP , Cleary P , Greene S , Mnich K , Almanza A , Chevet E , Dwyer RM , Oommen A , Legembre P *et al*., (2018) Inhibition of IRE1 RNase activity modulates the tumor cell secretome and enhances response to chemotherapy. Nat Commun 9, 3267. 30111846 10.1038/s41467-018-05763-8 PMC6093931

[51] Wang Y , Zong X , Mitra S , Mitra AK , Matei D & Nephew KP (2018) IL‐6 mediates platinum‐induced enrichment of ovarian cancer stem cells. JCI Insight 3, e122360. 10.1172/jci.insight.122360 PMC6328027 30518684

[52] Shen M , Dong C , Ruan X , Yan W , Cao M , Pizzo D , Wu X , Yang L , Liu L , Ren X *et al*., (2019) Chemotherapy‐induced extracellular vesicle miRNAs promote breast cancer stemness by targeting *ONECUT2* . Cancer Res 79, 3608–3621. 31118200 10.1158/0008-5472.CAN-18-4055 PMC8972808

[53] Guan X , LaPak KM , Hennessey RC , Yu CY , Shakya R , Zhang J & Burd CE (2017) Stromal senescence by prolonged CDK4/6 inhibition potentiates tumor growth. Mol Cancer Res 15, 237–249. 28039358 10.1158/1541-7786.MCR-16-0319 PMC5334447

[54] Gilbert LA & Hemann MT (2010) DNA damage‐mediated induction of a chemoresistant niche. Cell 143, 355–366. 21029859 10.1016/j.cell.2010.09.043 PMC2972353

[55] Obenauf AC , Zou Y , Ji AL , Vanharanta S , Shu W , Shi H , Kong X , Bosenberg MC , Wiesner T , Rosen N *et al*., (2015) Therapy‐induced tumour secretomes promote resistance and tumour progression. Nature 520, 368–372. 25807485 10.1038/nature14336 PMC4507807

[56] Yao Z , Murali B , Ren Q , Luo X , Faget DV , Cole T , Ricci B , Thotala D , Monahan J , van Deursen JM *et al*., (2020) Therapy‐induced senescence drives bone loss. Cancer Res 80, 1171–1182. 31932453 10.1158/0008-5472.CAN-19-2348 PMC7056549

[57] Baar MP , Brandt RMC , Putavet DA , Klein JDD , Derks KWJ , Bourgeois BRM , Stryeck S , Rijksen Y , van Willigenburg H , Feijtel DA *et al*., (2017) Targeted apoptosis of senescent cells restores tissue homeostasis in response to chemotoxicity and aging. Cell 169, 132–147.e116. 28340339 10.1016/j.cell.2017.02.031 PMC5556182

[58] Wu J , Qin H , Li T , Cheng K , Dong J , Tian M , Chai N , Guo H , Li J , You X *et al*., (2016) Characterization of site‐specific glycosylation of secreted proteins associated with multi‐drug resistance of gastric cancer. Oncotarget 7, 25315–25327. 27015365 10.18632/oncotarget.8287 PMC5041906

[59] Sharma A , Bender S , Zimmermann M , Riesterer O , Broggini‐Tenzer A & Pruschy MN (2016) Secretome signature identifies ADAM17 as novel target for radiosensitization of non‐small cell lung cancer. Clin Cancer Res 22, 4428–4439. 27076628 10.1158/1078-0432.CCR-15-2449

[60] Däbritz JHM , Yu Y , Milanovic M , Schönlein M , Rosenfeldt MT , Dörr JR , Kaufmann AM , Dörken B & Schmitt CA (2016) CD20‐targeting immunotherapy promotes cellular senescence in B‐cell lymphoma. Mol Cancer Ther 15, 1074–1081. 26880268 10.1158/1535-7163.MCT-15-0627

[61] Zhao B , Liu P , Fukumoto T , Nacarelli T , Fatkhutdinov N , Wu S , Lin J , Aird KM , Tang H‐Y , Liu Q *et al*., (2020) Topoisomerase 1 cleavage complex enables pattern recognition and inflammation during senescence. Nat Commun 11, 908. 32075966 10.1038/s41467-020-14652-y PMC7031389

[62] Hao X , Zhao B , Zhou W , Liu H , Fukumoto T , Gabrilovich D & Zhang R (2021) Sensitization of ovarian tumor to immune checkpoint blockade by boosting senescence‐associated secretory phenotype. iScience 24, 102016. 33490922 10.1016/j.isci.2020.102016 PMC7811168

[63] Jerby‐Arnon L , Shah P , Cuoco MS , Rodman C , Su MJ , Melms JC , Leeson R , Kanodia A , Mei S , Lin JR *et al*., (2018) A cancer cell program promotes T cell exclusion and resistance to checkpoint blockade. Cell 175, 984–997.e24. 30388455 10.1016/j.cell.2018.09.006 PMC6410377

[64] Luo X , Fu Y , Loza AJ , Murali B , Leahy KM , Ruhland MK , Gang M , Su X , Zamani A , Shi Y *et al*., (2016) Stromal‐initiated changes in the bone promote metastatic niche development. Cell Rep 14, 82–92. 26725121 10.1016/j.celrep.2015.12.016 PMC4706805

[65] Amor C , Feucht J , Leibold J , Ho YJ , Zhu C , Alonso‐Curbelo D , Mansilla‐Soto J , Boyer JA , Li X , Giavridis T *et al*., (2020) Senolytic CAR T cells reverse senescence‐associated pathologies. Nature 583, 127–132. 32555459 10.1038/s41586-020-2403-9 PMC7583560

[66] Santana‐Magal N , Farhat‐Younis L , Gutwillig A , Gleiberman A , Rasoulouniriana D , Tal L , Netanely D , Shamir R , Blau R , Feinmesser M *et al*., (2020) Melanoma‐secreted lysosomes trigger monocyte‐derived dendritic cell apoptosis and limit cancer immunotherapy. Cancer Res 80, 1942–1956. 32127354 10.1158/0008-5472.CAN-19-2944

[67] Pottier C , Fresnais M , Gilon M , Jerusalem G , Longuespee R & Sounni NE (2020) Tyrosine kinase inhibitors in cancer: breakthrough and challenges of targeted therapy. Cancers (Basel) 12, 731. 10.3390/cancers12030731 PMC7140093 32244867

[68] Goel S , DeCristo MJ , McAllister SS & Zhao JJ (2018) CDK4/6 inhibition in cancer: beyond cell cycle arrest. Trends Cell Biol 28, 911–925. 30061045 10.1016/j.tcb.2018.07.002 PMC6689321

[69] Finn RS , Dering J , Conklin D , Kalous O , Cohen DJ , Desai AJ , Ginther C , Atefi M , Chen I , Fowst C *et al*., (2009) PD 0332991, a selective cyclin D kinase 4/6 inhibitor, preferentially inhibits proliferation of luminal estrogen receptor‐positive human breast cancer cell lines in vitro. Breast Cancer Res 11, R77. 19874578 10.1186/bcr2419 PMC2790859

[70] Goel S , DeCristo MJ , Watt AC , BrinJones H , Sceneay J , Li BB , Khan N , Ubellacker JM , Xie S , Metzger‐Filho O *et al*., (2017) CDK4/6 inhibition triggers anti‐tumour immunity. Nature 548, 471–475. 28813415 10.1038/nature23465 PMC5570667

[71] Kovatcheva M , Liu DD , Dickson MA , Klein ME , O'Connor R , Wilder FO , Socci ND , Tap WD , Schwartz GK , Singer S *et al*., (2015) MDM2 turnover and expression of ATRX determine the choice between quiescence and senescence in response to CDK4 inhibition. Oncotarget 6, 8226–8243. 25803170 10.18632/oncotarget.3364 PMC4480747

[72] Wang B , Brandenburg S , Hernandez‐Segura A , van Vliet T , Jongbloed EM , Wilting S , Ohtani N , Jager A & Demaria M (2020) Pharmacological CDK4/6 inhibition unravels a p53‐induced secretory phenotype in senescent cells. bioRxiv 2020.06.05.135715. 10.1101/2020.06.05.135715

[73] Mastri M , Tracz A , Lee CR , Dolan M , Attwood K , Christensen JG , Liu S & Ebos JML (2018) A transient pseudosenescent secretome promotes tumor growth after antiangiogenic therapy withdrawal. Cell Rep 25, 3706–3720.e3708. 30590043 10.1016/j.celrep.2018.12.017 PMC13277708

[74] Wang X , Ai J , Liu H , Peng X , Chen H , Chen Y , Su Y , Shen A , Huang X , Ding J *et al*., (2019) The secretome engages STAT3 to favor a cytokine‐rich microenvironment in mediating acquired resistance to FGFR inhibitors. Mol Cancer Ther 18, 667–679. 30523050 10.1158/1535-7163.MCT-18-0179

[75] Liu S , Goldstein RH , Scepansky EM & Rosenblatt M (2009) Inhibition of rho‐associated kinase signaling prevents breast cancer metastasis to human bone. Cancer Res 69, 8742–8751. 19887617 10.1158/0008-5472.CAN-09-1541

[76] Rath N , Munro J , Cutiongco MF , Jagiello A , Gadegaard N , McGarry L , Unbekandt M , Michalopoulou E , Kamphorst JJ , Sumpton D *et al*., (2018) Rho kinase inhibition by AT13148 blocks pancreatic ductal adenocarcinoma invasion and tumor growth. Cancer Res 78, 3321–3336. 29669760 10.1158/0008-5472.CAN-17-1339 PMC6005347

[77] Vennin C , Chin VT , Warren SC , Lucas MC , Herrmann D , Magenau A , Melenec P , Walters SN , Del Monte‐Nieto G , Conway JR *et al*., (2017) Transient tissue priming via ROCK inhibition uncouples pancreatic cancer progression, sensitivity to chemotherapy, and metastasis. Sci Transl Med 9, eaai8504. 10.1126/scitranslmed.aai8504 28381539 PMC5777504

[78] Niklander S , Bandaru D , Lambert DW & Hunter KD (2020) ROCK inhibition modulates the senescence‐associated secretory phenotype (SASP) in oral keratinocytes. FEBS Open Bio 10, 2740–2749. 10.1002/2211-5463.13012 PMC7714064 33095981

[79] Huang A , Garraway LA , Ashworth A & Weber B (2020) Synthetic lethality as an engine for cancer drug target discovery. Nat Rev Drug Discov 19, 23–38. 31712683 10.1038/s41573-019-0046-z

[80] Sieben CJ , Sturmlechner I , van de Sluis B & van Deursen JM (2018) Two‐step senescence‐focused cancer therapies. Trends Cell Biol 28, 723–737. 29776716 10.1016/j.tcb.2018.04.006 PMC6102047

[81] Myrianthopoulos V , Evangelou K , Vasileiou PVS , Cooks T , Vassilakopoulos TP , Pangalis GA , Kouloukoussa M , Kittas C , Georgakilas AG & Gorgoulis VG (2019) Senescence and senotherapeutics: a new field in cancer therapy. Pharmacol Ther 193, 31–49. 30121319 10.1016/j.pharmthera.2018.08.006

[82] Short S , Fielder E , Miwa S & von Zglinicki T (2019) Senolytics and senostatics as adjuvant tumour therapy. EBioMedicine 41, 683–692. 30737084 10.1016/j.ebiom.2019.01.056 PMC6441870

[83] Zhu Y , Tchkonia T , Pirtskhalava T , Gower AC , Ding H , Giorgadze N , Palmer AK , Ikeno Y , Hubbard GB , Lenburg M *et al*., (2015) The Achilles' heel of senescent cells: from transcriptome to senolytic drugs. Aging Cell 14, 644–658. 25754370 10.1111/acel.12344 PMC4531078

[84] Gayle SS , Sahni JM , Webb BM , Weber‐Bonk KL , Shively MS , Spina R , Bar EE , Summers MK & Keri RA (2019) Targeting BCL‐xL improves the efficacy of bromodomain and extra‐terminal protein inhibitors in triple‐negative breast cancer by eliciting the death of senescent cells. J Biol Chem 294, 875–886. 30482844 10.1074/jbc.RA118.004712 PMC6341404

[85] Fleury H , Malaquin N , Tu V , Gilbert S , Martinez A , Olivier M‐A , Sauriol A , Communal L , Leclerc‐Desaulniers K & Carmona E (2019) Exploiting interconnected synthetic lethal interactions between PARP inhibition and cancer cell reversible senescence. Nat Commun 10, 1–15. 31186408 10.1038/s41467-019-10460-1 PMC6560032

[86] Guccini I , Revandkar A , D'Ambrosio M , Colucci M , Pasquini E , Mosole S , Troiani M , Brina D , Sheibani‐Tezerji R , Elia AR *et al*., (2021) Senescence reprogramming by TIMP1 deficiency promotes prostate cancer metastasis. Cancer Cell 39, 68–82.e9. 33186519 10.1016/j.ccell.2020.10.012

[87] Balakrishnan I , Danis E , Pierce A , Madhavan K , Wang D , Dahl N , Sanford B , Birks DK , Davidson N , Metselaar DS *et al*., (2020) Senescence induced by BMI1 inhibition is a therapeutic vulnerability in H3K27M‐mutant DIPG. Cell Rep 33, 108286. 33086074 10.1016/j.celrep.2020.108286 PMC7574900

[88] Helman A , Klochendler A , Azazmeh N , Gabai Y , Horwitz E , Anzi S , Swisa A , Condiotti R , Granit RZ , Nevo Y *et al*., (2016) p16(Ink4a)‐induced senescence of pancreatic beta cells enhances insulin secretion. Nat Med 22, 412–420. 26950362 10.1038/nm.4054 PMC5546206

[89] Hu Q , Peng J , Jiang L , Li W , Su Q , Zhang J , Li H , Song M , Cheng B , Xia J *et al*., (2020) Metformin as a senostatic drug enhances the anticancer efficacy of CDK4/6 inhibitor in head and neck squamous cell carcinoma. Cell Death Dis 11, 925. 33116117 10.1038/s41419-020-03126-0 PMC7595194

[90] Wang C , Vegna S , Jin H , Benedict B , Lieftink C , Ramirez C , de Oliveira RL , Morris B , Gadiot J , Wang W *et al*., (2019) Inducing and exploiting vulnerabilities for the treatment of liver cancer. Nature 574, 268–272. 31578521 10.1038/s41586-019-1607-3 PMC6858884

[91] Wiley CD , Liu S , Limbad C , Zawadzka AM , Beck J , Demaria M , Artwood R , Alimirah F , Lopez‐Dominguez JA , Kuehnemann C *et al*., (2019) SILAC analysis reveals increased secretion of hemostasis‐related factors by senescent cells. Cell Rep 28, 3329–3337.e5. 31553904 10.1016/j.celrep.2019.08.049 PMC6907691

[92] Demaria M , Ohtani N , Youssef SA , Rodier F , Toussaint W , Mitchell JR , Laberge RM , Vijg J , Van Steeg H , Dolle ME *et al*., (2014) An essential role for senescent cells in optimal wound healing through secretion of PDGF‐AA. Dev Cell 31, 722–733. 25499914 10.1016/j.devcel.2014.11.012 PMC4349629

